# Supplementary material for: COZOID: contact zone identifier for visual analysis of protein-protein interactions
Source: BMC Bioinformatics. 2018 Apr 6;19:125. doi: 10.1186/s12859-018-2113-6 (PMC5889581; doi:10.1186/s12859-018-2113-6)
Supplement: Supplementary file 2 — Software build. Executable binary file of the software tool. (ZIP 118,784 kb) [file 12859_2018_2113_MOESM2_ESM.zip › cozoid/platform/docs/testng-6.5.1-javadoc.zip!/org/testng/ITestNGMethod.html]

ITestNGMethod (TestNG)


---


|  |  |  |
| --- | --- | --- |
| **Overview** \| **Package** \| **Class** \| **Tree** \| **Deprecated** \| **Index** \| **Help** | |  |
| **PREV CLASS**   **NEXT CLASS** | **FRAMES**    **NO FRAMES**     **All Classes** |  |
| SUMMARY: NESTED \| FIELD \| CONSTR \| METHOD | DETAIL: FIELD \| CONSTR \| METHOD |  |


---


## org.testng Interface ITestNGMethod

**All Superinterfaces:**: java.lang.Cloneable, java.lang.Comparable, java.io.Serializable

---

``` public interface ITestNGMethod extends java.lang.Comparable, java.io.Serializable, java.lang.Cloneable ```

Describes a TestNG annotated method and the instance on which it will be invoked.
This interface is not meant to be implemented by users.

**Author:**
:   Cedric Beust, May 3, 2004

---

| **Method Summary** | |
| --- | --- |
| `void` | `addFailedInvocationNumber(int number)`             The list of invocation numbers that failed, which is only applicable for methods that have a data provider. |
| `void` | `addMethodDependedUpon(java.lang.String methodName)` |
| `boolean` | `canRunFromClass(IClass testClass)`             Returns if this ITestNGMethod can be invoked from within IClass. |
| `ITestNGMethod` | `clone()` |
| `java.util.Map<java.lang.String,java.lang.String>` | `findMethodParameters(XmlTest test)` |
| `java.lang.String[]` | `getAfterGroups()` |
| `java.lang.String[]` | `getBeforeGroups()`             Before and After groups |
| `org.testng.internal.ConstructorOrMethod` | `getConstructorOrMethod()` |
| `int` | `getCurrentInvocationCount()` |
| `long` | `getDate()` |
| `java.lang.String` | `getDescription()` |
| `boolean` | `getEnabled()` |
| `java.util.List<java.lang.Integer>` | `getFailedInvocationNumbers()` |
| `java.lang.String[]` | `getGroups()` |
| `java.lang.String[]` | `getGroupsDependedUpon()` |
| `java.lang.String` | `getId()` |
| `java.lang.Object` | `getInstance()` |
| `long[]` | `getInstanceHashCodes()`             Needed for serialization. |
| `java.lang.Object[]` | `getInstances()`             **Deprecated.** *Use getInstance().* |
| `int` | `getInvocationCount()` |
| `java.util.List<java.lang.Integer>` | `getInvocationNumbers()`             Which invocation numbers of this method should be used (only applicable if it uses a data provider). |
| `long` | `getInvocationTimeOut()`             The time under which all invocationCount methods need to complete by. |
| `java.lang.reflect.Method` | `getMethod()`             **Deprecated.** *This method is deprecated and can now return null. Use getConstructorOrMethod() instead.* |
| `java.lang.String` | `getMethodName()`             Returns the method name. |
| `java.lang.String[]` | `getMethodsDependedUpon()` |
| `java.lang.String` | `getMissingGroup()`             If a group was not found. |
| `int` | `getParameterInvocationCount()` |
| `int` | `getPriority()`             The scheduling priority. |
| `java.lang.Class` | `getRealClass()` |
| `IRetryAnalyzer` | `getRetryAnalyzer()` |
| `int` | `getSuccessPercentage()` |
| `ITestClass` | `getTestClass()` |
| `int` | `getThreadPoolSize()` |
| `long` | `getTimeOut()` |
| `XmlTest` | `getXmlTest()` |
| `boolean` | `ignoreMissingDependencies()` |
| `void` | `incrementCurrentInvocationCount()` |
| `boolean` | `isAfterClassConfiguration()` |
| `boolean` | `isAfterGroupsConfiguration()` |
| `boolean` | `isAfterMethodConfiguration()` |
| `boolean` | `isAfterSuiteConfiguration()` |
| `boolean` | `isAfterTestConfiguration()` |
| `boolean` | `isAlwaysRun()` |
| `boolean` | `isBeforeClassConfiguration()` |
| `boolean` | `isBeforeGroupsConfiguration()` |
| `boolean` | `isBeforeMethodConfiguration()` |
| `boolean` | `isBeforeSuiteConfiguration()` |
| `boolean` | `isBeforeTestConfiguration()` |
| `boolean` | `isTest()` |
| `void` | `setDate(long date)` |
| `void` | `setId(java.lang.String id)` |
| `void` | `setIgnoreMissingDependencies(boolean ignore)` |
| `void` | `setInvocationCount(int count)` |
| `void` | `setInvocationNumbers(java.util.List<java.lang.Integer> numbers)` |
| `void` | `setMissingGroup(java.lang.String group)` |
| `void` | `setParameterInvocationCount(int n)` |
| `void` | `setPriority(int priority)` |
| `void` | `setRetryAnalyzer(IRetryAnalyzer retryAnalyzer)` |
| `void` | `setSkipFailedInvocations(boolean skip)` |
| `void` | `setTestClass(ITestClass cls)`             Sets the test class having this method. |
| `void` | `setThreadPoolSize(int threadPoolSize)` |
| `void` | `setTimeOut(long timeOut)` |
| `boolean` | `skipFailedInvocations()` |

**Methods inherited from interface java.lang.Comparable**

`compareTo`

**Method Detail**

### getRealClass

```
java.lang.Class getRealClass()
```

:   **Returns:**: The real class on which this method was declared (can be different from getMethod().getDeclaringClass() if the test method was defined in a superclass).

---


### getTestClass

```
ITestClass getTestClass()
```

---


### setTestClass

```
void setTestClass(ITestClass cls)
```

:   Sets the test class having this method. This is not necessarily the declaring class.

    :   **Parameters:**: `cls` - The test class having this method.

---


### getMethod

```
@Deprecated
java.lang.reflect.Method getMethod()
```

:   **Deprecated.** *This method is deprecated and can now return null. Use
    getConstructorOrMethod() instead.*

    :   **Returns:**: the corresponding Java test method.

---


### getMethodName

```
java.lang.String getMethodName()
```

:   Returns the method name. This is needed for serialization because
    methods are not Serializable.

    :   **Returns:**: the method name.

---


### getInstances

```
@Deprecated
java.lang.Object[] getInstances()
```

:   **Deprecated.** *Use getInstance().*

    :   **Returns:**: All the instances the methods will be invoked upon. This will typically be an array of one object in the absence of an @Factory annotation.

---


### getInstance

```
java.lang.Object getInstance()
```

---


### getInstanceHashCodes

```
long[] getInstanceHashCodes()
```

:   Needed for serialization.

---


### getGroups

```
java.lang.String[] getGroups()
```

:   **Returns:**: The groups this method belongs to, possibly added to the groups declared on the class.

---


### getGroupsDependedUpon

```
java.lang.String[] getGroupsDependedUpon()
```

:   **Returns:**: The groups this method depends on, possibly added to the groups declared on the class.

---


### getMissingGroup

```
java.lang.String getMissingGroup()
```

:   If a group was not found.

---


### setMissingGroup

```
void setMissingGroup(java.lang.String group)
```

---


### getBeforeGroups

```
java.lang.String[] getBeforeGroups()
```

:   Before and After groups

---


### getAfterGroups

```
java.lang.String[] getAfterGroups()
```

---


### getMethodsDependedUpon

```
java.lang.String[] getMethodsDependedUpon()
```

:   **Returns:**: The methods this method depends on, possibly added to the methods declared on the class.

---


### addMethodDependedUpon

```
void addMethodDependedUpon(java.lang.String methodName)
```

---


### isTest

```
boolean isTest()
```

:   **Returns:**: true if this method was annotated with @Test

---


### isBeforeMethodConfiguration

```
boolean isBeforeMethodConfiguration()
```

:   **Returns:**: true if this method was annotated with @Configuration and beforeTestMethod = true

---


### isAfterMethodConfiguration

```
boolean isAfterMethodConfiguration()
```

:   **Returns:**: true if this method was annotated with @Configuration and beforeTestMethod = false

---


### isBeforeClassConfiguration

```
boolean isBeforeClassConfiguration()
```

:   **Returns:**: true if this method was annotated with @Configuration and beforeClassMethod = true

---


### isAfterClassConfiguration

```
boolean isAfterClassConfiguration()
```

:   **Returns:**: true if this method was annotated with @Configuration and beforeClassMethod = false

---


### isBeforeSuiteConfiguration

```
boolean isBeforeSuiteConfiguration()
```

:   **Returns:**: true if this method was annotated with @Configuration and beforeSuite = true

---


### isAfterSuiteConfiguration

```
boolean isAfterSuiteConfiguration()
```

:   **Returns:**: true if this method was annotated with @Configuration and afterSuite = true

---


### isBeforeTestConfiguration

```
boolean isBeforeTestConfiguration()
```

:   **Returns:**: true if this method is a @BeforeTest (@Configuration beforeTest=true)

---


### isAfterTestConfiguration

```
boolean isAfterTestConfiguration()
```

:   **Returns:**: true if this method is an @AfterTest (@Configuration afterTest=true)

---


### isBeforeGroupsConfiguration

```
boolean isBeforeGroupsConfiguration()
```

---


### isAfterGroupsConfiguration

```
boolean isAfterGroupsConfiguration()
```

---


### getTimeOut

```
long getTimeOut()
```

:   **Returns:**: The timeout in milliseconds.

---


### setTimeOut

```
void setTimeOut(long timeOut)
```

---


### getInvocationCount

```
int getInvocationCount()
```

:   **Returns:**: the number of times this method needs to be invoked.

---


### setInvocationCount

```
void setInvocationCount(int count)
```

---


### getSuccessPercentage

```
int getSuccessPercentage()
```

:   **Returns:**: the success percentage for this method (between 0 and 100).

---


### getId

```
java.lang.String getId()
```

:   **Returns:**: The id of the thread this method was run in.

---


### setId

```
void setId(java.lang.String id)
```

---


### getDate

```
long getDate()
```

---


### setDate

```
void setDate(long date)
```

---


### canRunFromClass

```
boolean canRunFromClass(IClass testClass)
```

:   Returns if this ITestNGMethod can be invoked from within IClass.

---


### isAlwaysRun

```
boolean isAlwaysRun()
```

:   **Returns:**: true if this method is alwaysRun=true

---


### getThreadPoolSize

```
int getThreadPoolSize()
```

:   **Returns:**: the number of threads to be used when invoking the method on parallel

---


### setThreadPoolSize

```
void setThreadPoolSize(int threadPoolSize)
```

---


### getEnabled

```
boolean getEnabled()
```

---


### getDescription

```
java.lang.String getDescription()
```

---


### incrementCurrentInvocationCount

```
void incrementCurrentInvocationCount()
```

---


### getCurrentInvocationCount

```
int getCurrentInvocationCount()
```

---


### setParameterInvocationCount

```
void setParameterInvocationCount(int n)
```

---


### getParameterInvocationCount

```
int getParameterInvocationCount()
```

---


### clone

```
ITestNGMethod clone()
```

---


### getRetryAnalyzer

```
IRetryAnalyzer getRetryAnalyzer()
```

---


### setRetryAnalyzer

```
void setRetryAnalyzer(IRetryAnalyzer retryAnalyzer)
```

---


### skipFailedInvocations

```
boolean skipFailedInvocations()
```

---


### setSkipFailedInvocations

```
void setSkipFailedInvocations(boolean skip)
```

---


### getInvocationTimeOut

```
long getInvocationTimeOut()
```

:   The time under which all invocationCount methods need to complete by.

---


### ignoreMissingDependencies

```
boolean ignoreMissingDependencies()
```

---


### setIgnoreMissingDependencies

```
void setIgnoreMissingDependencies(boolean ignore)
```

---


### getInvocationNumbers

```
java.util.List<java.lang.Integer> getInvocationNumbers()
```

:   Which invocation numbers of this method should be used (only applicable
    if it uses a data provider). If this value is an empty list, use all the values
    returned from the data provider. These values are read from the XML file in
    the  tag.

---


### setInvocationNumbers

```
void setInvocationNumbers(java.util.List<java.lang.Integer> numbers)
```

---


### addFailedInvocationNumber

```
void addFailedInvocationNumber(int number)
```

:   The list of invocation numbers that failed, which is only applicable for
    methods that have a data provider.

---


### getFailedInvocationNumbers

```
java.util.List<java.lang.Integer> getFailedInvocationNumbers()
```

---


### getPriority

```
int getPriority()
```

:   The scheduling priority. Lower priorities get scheduled first.

---


### setPriority

```
void setPriority(int priority)
```

---


### getXmlTest

```
XmlTest getXmlTest()
```

:   **Returns:**: the XmlTest this method belongs to.

---


### getConstructorOrMethod

```
org.testng.internal.ConstructorOrMethod getConstructorOrMethod()
```

---


### findMethodParameters

```
java.util.Map<java.lang.String,java.lang.String> findMethodParameters(XmlTest test)
```

:   **Parameters:**: `test` - **Returns:**: the parameters found in the include tag, if any


---


|  |  |  |
| --- | --- | --- |
| **Overview** \| **Package** \| **Class** \| **Tree** \| **Deprecated** \| **Index** \| **Help** | |  |
| **PREV CLASS**   **NEXT CLASS** | **FRAMES**    **NO FRAMES**     **All Classes** |  |
| SUMMARY: NESTED \| FIELD \| CONSTR \| METHOD | DETAIL: FIELD \| CONSTR \| METHOD |  |


---
